# Life’s Crucial 9 score and chronic kidney disease: insights from NHANES 2005–2018 and the mediating role of systemic inflammation and oxidative stress

**DOI:** 10.3389/fmed.2025.1605931

**Published:** 2025-06-18

**Authors:** Shiyuan Qin, Jin Yang, Zili Wang, Pinglin He, Taotao Dong, Zhengyun Ren, Qiuyang Li

**Affiliations:** ^1^Department of Urology, The Affiliated Hospital and Clinical Medical College of Chengdu University, Chengdu, Sichuan, China; ^2^College of Medicine, Southwest Jiaotong University, Chengdu, Sichuan, China; ^3^Department of Pulmonary and Critical Care Medicine, The Affiliated Hospital and Clinical Medical College of Chengdu University, Chengdu, Sichuan, China

**Keywords:** CKD, LC9, LE8, mediation analysis, NHANES

## Abstract

**Background:**

Chronic kidney disease (CKD) is a growing global health burden, closely linked to metabolic and cardiovascular risk factors. Life’s Crucial 9 (LC9) is a novel health assessment tool that expands upon Life’s Essential 8 (LE8) by incorporating mental health (depression) as a key component. This study aimed to investigate the association between LC9 and CKD, compare its predictive value with LE8, and explore potential mediating mechanisms.

**Methods:**

This study analyzed data from 16,431 participants in the National Health and Nutrition Examination Survey (NHANES) 2005–2018. Logistic regression models were used to assess the association between LC9 and CKD, with comparisons to LE8. Restricted cubic spline models were applied to explore potential nonlinear relationships. Mediation analysis was conducted to evaluate whether systemic inflammation and oxidative stress mediated the association between LC9 and CKD. Receiver operating characteristic (ROC) analysis was performed to compare the predictive performance of LC9 and LE8 for CKD risk.

**Results:**

Higher LC9 scores were significantly associated with a lower risk of CKD in both continuous and quartile-based analyses. A nonlinear relationship was observed between LC9 and CKD risk (*P* for nonlinearity < 0.001). Mediation analysis indicated that systemic immune-inflammation index (SII) and uric acid partially mediated the association between LC9 and CKD, with mediation proportions of 3.32 and 11.13%, respectively. ROC analysis showed that LC9 and LE8 had comparable predictive abilities for CKD.

**Conclusion:**

Higher LC9 scores are associated with a reduced risk of CKD, with systemic inflammation and uric acid levels partially mediating this relationship. These findings highlight the importance of comprehensive lifestyle and mental health interventions in CKD prevention and management.

## 1 Introduction

Cardiovascular health (CVH) metrics have undergone significant evolution since the American Heart Association (AHA) first introduced Life’s Simple 7 (LS7) in 2010 (1, 2). This framework, comprising seven modifiable factors—diet, physical activity (PA), smoking, body mass index (BMI), blood pressure, blood glucose, and total cholesterol—aimed to standardize cardiovascular risk assessment and promote preventive strategies (2). LS7 demonstrated robust associations with reduced risks of cardiovascular disease (CVD), diabetes, and mortality, establishing itself as a cornerstone in public health initiatives (2–4). However, emerging evidence highlighted gaps in its comprehensiveness, particularly regarding sleep health and refined scoring algorithms. In response, the AHA unveiled Life’s Essential 8 (LE8) in 2020, integrating sleep health as an eighth component and revising scoring methodologies (5). While maintaining continuity with LS7’s core principles, LE8 expanded the operationalization of CVH by incorporating sleep metrics, subsequently demonstrating associations with a broader spectrum of health outcomes—from nonalcoholic fatty liver disease to all-cause mortality—in population-based studies (6, 7).

The application of LE8 has extended beyond cardiovascular outcomes to chronic conditions such as chronic kidney disease (CKD), a condition intricately linked to cardiovascular pathology (8, 9). CKD and CVD share overlapping risk factors, including hypertension, atherosclerosis, diabetes, and systemic inflammation (10, 11). For example, atherosclerosis is one of the leading causes of morbidity and mortality in CKD and is also closely involved in the onset and progression of CVD (11, 12). Recent studies leveraging LE8 have demonstrated its inverse association with CKD incidence, suggesting that optimal CVH metrics may mitigate renal injury through shared pathways (8). For instance, adherence to LE8’s dietary and PA guidelines may improve blood pressure regulation and glucose metabolism, both well-established contributors to CKD progression (13, 14). Despite these advances, the exclusion of psychological health from LE8 has sparked debate. Mental well-being, particularly depression, is increasingly recognized as an important influencing factor of cardiovascular and renal outcomes, yet its integration into CVH metrics remains unresolved (15, 16).

This gap prompted the conceptualization of Life’s Crucial 9 (LC9), an exploratory framework combining LE8 with psychological health, operationalized through depression screening tools like the Patient Health Questionnaire-9 (PHQ-9) (17). Although not yet formally endorsed by the AHA, LC9 builds on accumulating evidence linking depression to adverse cardiometabolic outcomes. Depression affects over 300 million individuals globally and is associated with maladaptive health behaviors and physiological dysregulation (18). Notably, the prevalence of depression among patients with CKD is high and closely related to poor prognosis (19). Despite these insights, no studies have evaluated whether incorporating depression into CVH metrics enhances CKD risk prediction compared to LE8 alone.

Furthermore, the biological pathways connecting CVH metrics to CKD remain underexplored. Oxidative stress and inflammation are established mediators of renal injury, driving glomerulosclerosis and tubular atrophy (20, 21). At the same time, oxidative stress and inflammation also play critical roles in the pathogenesis and progression of CVD, and targeting these pathways has been shown to be an effective strategy for the prevention and treatment of CVD (22, 23). LC9’s components, such as healthy diet and PA, may attenuate these pathways by reducing oxidative stress and inflammation (24, 25). Similarly, depression is associated with elevated oxidative stress and systemic inflammatory, suggesting that LC9’s inclusion of psychological health may offer a more holistic representation of CKD risk mechanisms (26, 27). However, whether LE8 or LC9 exerts protective effects on CKD through these mediators remains unexamined.

To address these knowledge gaps, this study utilized data from the National Health and Nutrition Examination Survey (NHANES) (2005–2018) to achieve three objectives: (1) compare predictive accuracy of LE8 and LC9 for CKD risk, (2) evaluate the incremental predictive value of depression beyond LE8 for CKD risk, and (3) investigate the mediating roles of oxidative stress (bilirubin; uric acid) and systemic inflammation (systemic immune-inflammation index, SII; systemic inflammation response index, SIRI) in these relationships. This study examines how oxidative stress and systemic inflammation mediate the link between LC9 scores and CKD risk, identifying modifiable pathways for prevention.

## 2 Methods

### 2.1 Study design and participants

This cross-sectional study utilized data from the NHANES, a nationally representative program conducted by the National Center for Health Statistics (NCHS) to assesses the health status of the non-institutionalized U.S. residents.

**Figure 1:**
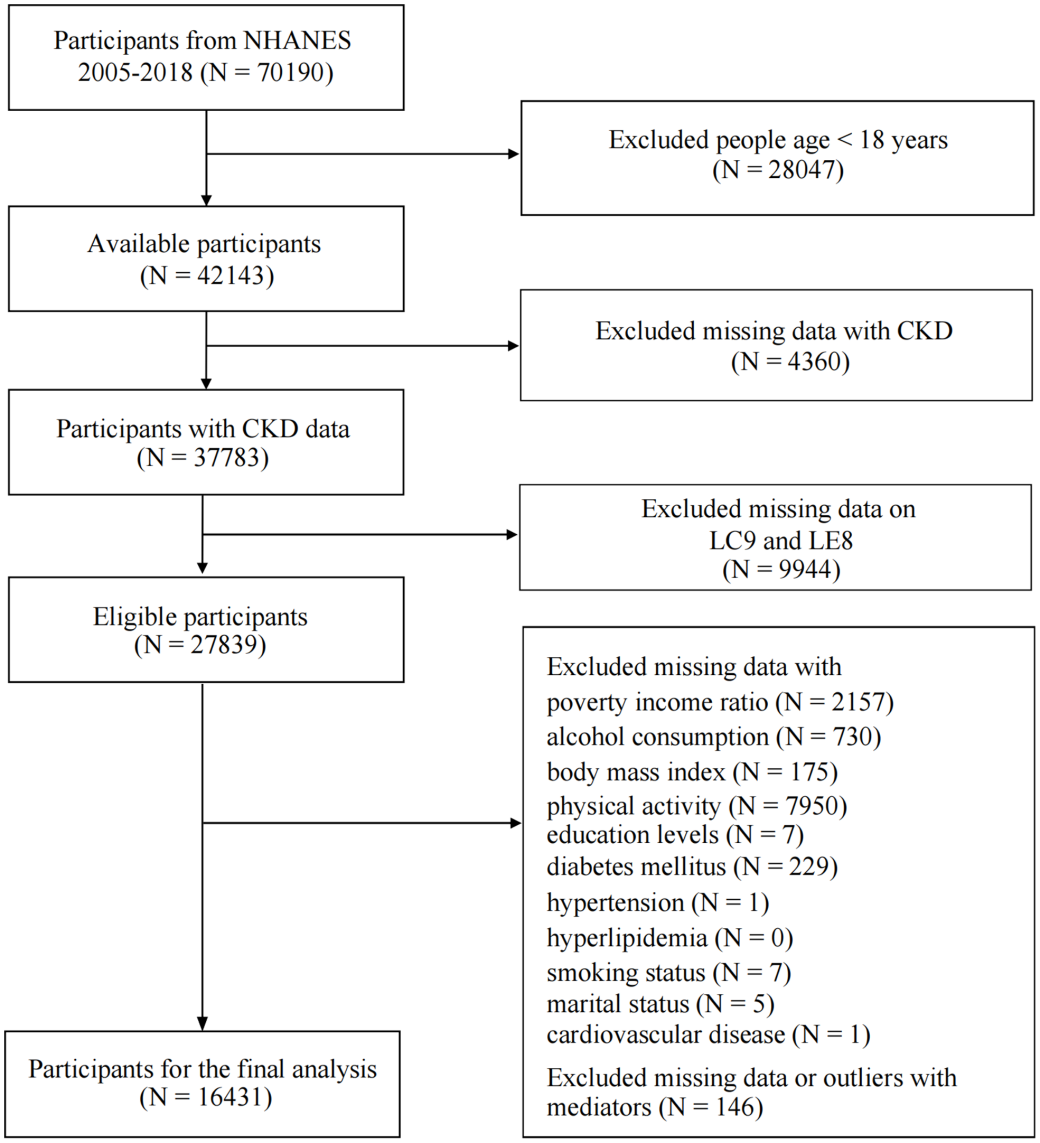
Flowchart of procedures for participants selection and inclusion. CKD, chronic kidney disease; LC9, Life’s Crucial 9; LE8, Life’s Essential 8.

We analyzed seven consecutive cycles from 2005 to 2018. The decision to focus on this period was based on the following considerations: (1) The necessary variables required for calculating LC9 became fully accessible starting in the 2005 cycle. (2) Excluding the 2019–2020 cycle ensured consistency in data collection protocols, avoiding potential biases introduced by disruptions related to the COVID-19 pandemic. Initially, 70,190 participants were included in the dataset. However, after excluding individuals who were under 18 years old, had missing data, or presented abnormal blood cell values, a final sample of 16,431 participants remained for analysis. Supplementary Figure S1 presents box plots illustrating the distribution of lymphocytes, monocytes, neutrophils, and platelets, highlighting outlier identification and exclusion steps undertaken to improve data reliability. The detailed flowchart of the inclusion and exclusion process is presented in Figure 1. By applying survey weights, our final study population represents an estimated 132,069,346 weighted individuals in the U.S. population.

### 2.2 Ethics statement

This study was carried out under the guidance of the NCHS and received ethical approval from the NCHS Institutional Review Board (IRB). Informed consent was obtained from all eligible participants before data collection and health assessments, ensuring adherence to ethical research principles and regulatory standards.

### 2.3 Definitions of LC9 and LE8

The LC9 score is a composite measure of cardiovascular and mental health, integrating eight key CVH metrics along with a depression assessment. It builds upon the LE8 framework while incorporating mental well-being as a crucial component of overall health.

The LC9 score consists of four health behaviors and four health factors, supplemented by a depression metric. Health behaviors include diet quality, nicotine exposure, PA and sleep duration. Health factors include BMI, blood pressure, blood glucose and blood lipids. The inclusion of depression is based on growing evidence linking mental health to cardiometabolic outcomes and chronic disease progression (5, 17). Diet quality was evaluated using the Healthy Eating Index (HEI) 2015, which measures adherence to dietary guidelines. Dietary data were obtained from two 24-h dietary recalls and analyzed using the U.S. Department of Agriculture’s food pattern equivalents database. The HEI-2015 was computed using an established algorithm provided by the National Cancer Institute. The data of nicotine exposure, PA and sleep duration were assessed through standardized self-reported questionnaires. BMI was calculated as weight (kg) divided by height (m^2^), with classifications based on standard BMI categories. Blood pressure was measured during standardized physical examinations. Blood glucose and blood lipids were assessed thought blood samples analyzed in centralized laboratories. Depression score was assessed using the PHQ-9, a validated tool for screening depressive symptoms.

Each of the eight CVH metrics was scored on a continuous scale from 0 to 100 points, based on a modified Delphi approach that aligns with expert consensus on health risk stratification (28). The LC9 score was then calculated as the mean of the eight LE8 components plus the depression score, ensuring that mental health was integrated into the overall assessment (5). In contrast, the LE8 score was calculated without considering the depression component. This expanded framework allows LC9 to serve as a more holistic health index that captures both physical and psychological dimensions of chronic disease risk. While the LC9 score is conceptually grounded in the LE8 framework and supported by emerging literature on mental health integration, it remains an exploratory construct. To date, LC9 has not undergone formal independent validation. Nevertheless, a growing body of research has demonstrated its potential utility in predicting a broad spectrum of health outcomes, including cardiovascular events, biological ageing, and all-cause mortality (5, 29). These findings suggest that LC9 may offer a more comprehensive and integrative approach to health risk assessment, particularly by capturing psychological dimensions that are not reflected in LE8 alone.

Notably, according to the referenced literature, both fasting and non-fasting blood samples can be used to assess blood glucose and blood lipids, with slight variations in calculation methods (28). In this study, we utilized non-fasting blood samples for these measurements.

### 2.4 Definitions of CKD

CKD is diagnosed when structural or functional kidney abnormalities persist for at least 3 months (30). This condition is identified by either a reduced estimated glomerular filtration rate (eGFR) of less than 60 mL/min/1.73m^2^, an increased urinary albumin-to-creatinine ratio (UACR) of 30 mg/g or higher, or both (8). The Chronic Kidney Disease Epidemiology Collaboration (CKD-EPI) equation, which relies on serum creatinine levels, is commonly used to estimate eGFR. Between 2005 and 2016, the NHANES study assessed serum and urinary creatinine levels using the Jaffe rate method. In contrast, for the 2017–2018 cycle, these measurements were conducted using enzymatic assays. Urinary albumin concentrations were determined through a fluorescence-based immunoassay.

### 2.5 Definition of potential mediators

In this study, we selected four potential mediators to represent two key biological processes: systemic inflammatory (SIRI and SII) and oxidative stress (bilirubin and uric acid). Each mediator is described in detail below. Both SIRI and SII serve as biomarkers for systemic inflammation (31). SIRI = (neutrophil count × monocyte count) / lymphocyte count, and SII = (platelet count × neutrophil count) /lymphocyte count. Bilirubin is a metabolic byproduct of hemoglobin breakdown and plays a dual role as both an antioxidant and a marker of liver function. Changes in bilirubin levels have been associated with increased oxidative stress and disturbances in metabolic homeostasis (32). Uric acid, another metabolic byproduct, originates from purine metabolism and serves as a key indicator of metabolic health (33).

### 2.6 Definition of covariates

Detailed descriptions of data collection and classification methods for hyperlipidemia, hypertension, and diabetes mellitus (DM) are provided in Supplementary Table S1, while the definition of cardiovascular disease (CVD) follows criteria established in prior research (34). Participants completed a PA questionnaire that captured details of activities performed over the past 30 days. The questionnaire documented activity type, frequency, and intensity, classifying them into moderate and vigorous activity levels based on their physiological impact (35, 36). Moderate-intensity activities were those causing a slight increase in breathing and heart rate, whereas vigorous-intensity activities led to substantial elevations in both parameters. The total physical activity volume (PA total MET) was estimated by summing the Metabolic Equivalent (MET) scores from activities related to work, recreation, and transportation. Age and Poverty-Income Ratio (PIR) were converted into categorical variables according to the classification scheme outlined in Table 1. Further details regarding the definitions and classifications of covariates can be found in previously published studies (31).

**Table 1:** Descriptive characteristics of the study population stratified by CKD.

Characteristic	Total	Non-CKD	CKD	*p*-values
	(*N* = 16,431)	(*N* = 14,192)	(*N* = 2,239)	
LC9	74.05 ± 0.23	74.69 ± 0.23	68.73 ± 0.54	**< 0.001**
LE8	72.03 ± 0.24	72.70 ± 0.24	66.44 ± 0.55	**< 0.001**
Bilirubin (mg/dl)	0.67 ± 0.01	0.68 ± 0.01	0.65 ± 0.01	
Uric acid (mg/dl)	5.44 ± 0.02	5.39 ± 0.02	5.90 ± 0.06	**< 0.001**
SII	526.30 ± 4.18	519.74 ± 4.41	580.38 ± 9.91	**< 0.001**
SIRI	1.22 ± 0.01	1.19 ± 0.01	1.46 ± 0.04	**< 0.001**
PA total MET (MET)	4570.2 ± 95.9	4687.8 ± 104.9	3600.1 ± 176.3	**< 0.001**
Age, *n* (%)				**< 0.001**
18–29	3,037 (20.37)	2,886 (95.06)	151 (4.94)	
30–44	4,452 (28.15)	4,138 (93.58)	314 (6.42)	
45–59	4,198 (29.18)	3,749 (91.24)	449 (8.76)	
> =60	4,744 (22.31)	3,419 (75.62)	1,325 (24.38)	
Sex, *n* (%)				**< 0.001**
Female	7,867 (48.28)	6,760 (87.82)	1,107 (12.18)	
Male	8,564 (51.72)	7,432 (90.47)	1,132 (9.53)	
Race/Ethnicity, *n* (%)				**< 0.001**
Non-Hispanic white	7,649 (70.21)	6,588 (89.15)	1,061 (10.85)	
Non-Hispanic black	3,233 (9.63)	2,655 (85.87)	578 (14.13)	
Hispanic	3,780 (12.97)	3,363 (91.34)	417 (8.66)	
other race	1769 (7.18)	1,586 (90.19)	183 (9.81)	
BMI, *n* (%)				**< 0.001**
Underweight/Normal	4,858 (31.24)	4,284 (90.74)	574 (9.26)	
Overweight	5,510 (33.79)	4,827 (90.60)	683 (9.40)	
Obese	6,063 (34.97)	5,081 (86.45)	982 (13.55)	
Marital status, *n* (%)				**< 0.001**
Married/Living with Partner	10,002 (63.46)	8,743 (89.81)	1,259 (10.19)	
Never married	3,208 (20.12)	2,930 (93.66)	278 (6.34)	
Widowed/Divorced/Separated	3,221 (16.43)	2,519 (81.32)	702 (18.68)	
PIR, *n* (%)				**< 0.001**
< 1.3	4,688 (19.51)	3,989 (87.51)	699 (12.49)	
1.3–3.5	6,089 (33.93)	5,159 (87.41)	930 (12.59)	
> 3.5	5,654 (46.57)	5,044 (91.19)	610 (8.81)	
Education levels, *n* (%)				**< 0.001**
Below high school	1,084 (3.22)	881 (82.40)	203 (17.60)	
High school	5,584 (30.27)	4,698 (86.88)	886 (13.12)	
Above high school	9,763 (66.51)	8,613 (90.57)	1,150 (9.43)	
Alcohol consumption, *n* (%)				**< 0.001**
Never	1946 (9.46)	1,628 (86.97)	318 (13.03)	
Former	2,289 (11.05)	1805 (83.20)	484 (16.80)	
Mild	5,959 (38.01)	5,174 (89.18)	785 (10.82)	
Moderate	2,784 (18.86)	2,477 (90.48)	307 (9.52)	
Heavy	3,453 (22.61)	3,108 (91.99)	345 (8.01)	
Smoking status, *n* (%)				
Never	9,192 (56.73)	8,068 (90.00)	1,124 (10.00)	
Former	4,001 (24.46)	3,291 (86.53)	710 (13.47)	
Now	3,238 (18.81)	2,833 (90.21)	405 (9.79)	
Hyperlipidemia, *n* (%)				**< 0.001**
No	4,935 (31.13)	4,516 (93.12)	419 (6.88)	
Yes	11,496 (68.87)	9,676 (87.42)	1820 (12.58)	
Hypertension, *n* (%)				**< 0.001**
No	10,047 (66.35)	9,308 (93.80)	739 (6.20)	
Yes	6,384 (33.65)	4,884 (80.11)	1,500 (19.89)	
Diabetes mellitus, *n* (%)				**< 0.001**
No	12,518 (81.45)	11,319 (91.89)	1,199 (8.11)	
Prediabetes	1,369 (7.35)	1,151 (86.96)	218 (13.04)	
DM	2,544 (11.21)	1722 (71.07)	822 (28.93)	
CVD, *n* (%)				**< 0.001**
No	15,024 (93.22)	13,280 (90.69)	1744 (9.31)	
Yes	1,407 (6.78)	912 (68.57)	495 (31.43)	

CKD, Chronic kidney disease; LC9, Life’s Crucial 9; LE8, Life’s Essential 8; GGT, Serum gamma-glutamyltransferase; SII, Systemic immune-inflammation index; SIRI, Systemic inflammation response index; MET, Metabolic Equivalent; BMI, Body mass index; PIR, Poverty income ratio; CVD, Cardiovascular disease. Bold values indicate statistical significance at *P* < 0.05.

### 2.7 Statistical analyses

For this study, we utilized data spanning the 2005 to 2018 NHANES survey cycles, incorporating a total of seven consecutive cycles. Following the analytical guidelines provided on the NHANES official website, appropriate survey weights were applied to ensure accurate statistical analyses. Descriptive statistics were used to summarize the characteristics of study participants. Continuous variables were expressed as means with standard deviations (SD), while categorical variables were presented as frequencies and percentages. To compare differences between groups based on CKD status, Chi-square tests were used for categorical variables, and analysis of variance (ANOVA) was applied for continuous variables. Before conducting regression analyses, collinearity diagnostics were performed on all covariates. Variance inflation factors (VIFs) were calculated to assess multicollinearity, with all covariates yielding VIFs values below 3, suggesting no significant collinearity issues. All statistical analyses were carried out in R Studio (version 4.3.1) using the nhanesR package (version 0.9.4.3), adhering to the STROBE guidelines.

The LC9 and LE8 scores were categorized into quartiles (Q1–Q4), ranging from the lowest to highest levels, as outlined in Supplementary Table S2. To explore the relationship between LC9 and CKD, weighted logistic regression models were implemented. Associations were reported as odds ratios (ORs) with 95% confidence intervals (CIs). To verify the assumptions of logistic regression, the linearity between continuous independent variables and the logit(p) transformation was examined. Subgroup analyses were conducted to determine whether the relationship between LC9 and CKD varied across different population strata, such as age, sex, and BMI categories. This approach ensured that findings remained consistent across different demographic and clinical groups. Sensitivity analyses were also performed by conducting regressions without applying survey weights to assess the robustness of the results.

**Figure 2:**
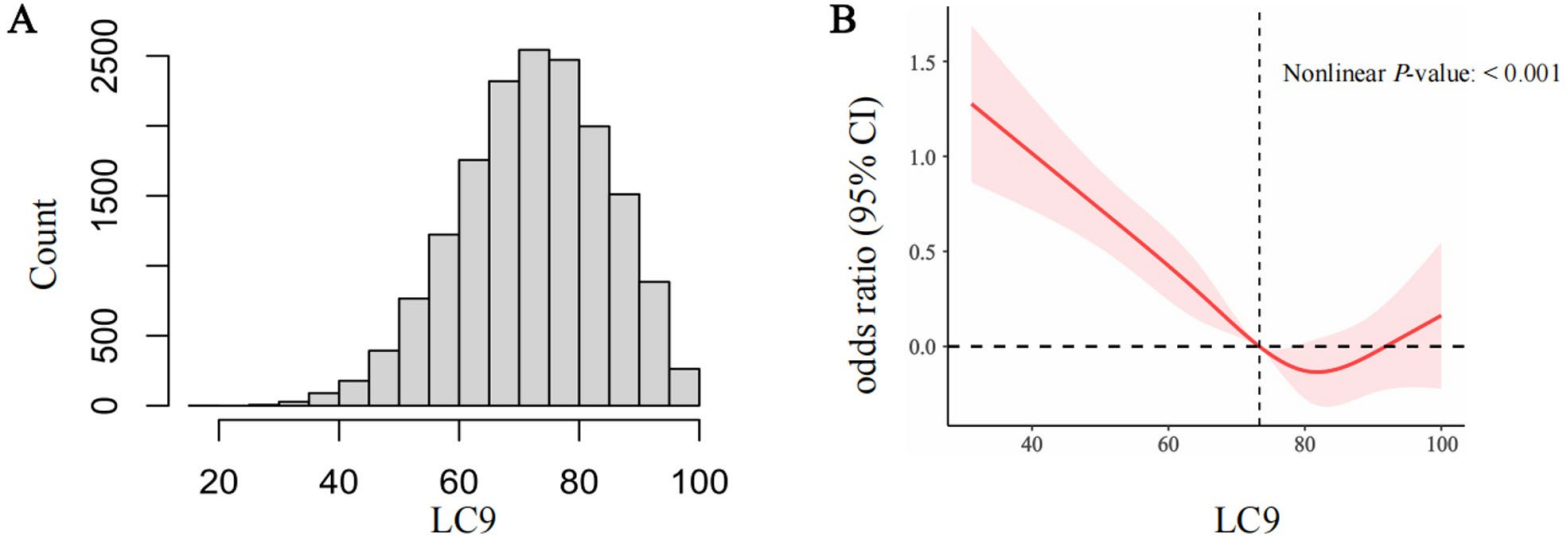
The distribution of LC9 **(A)**. The full-adjusted relationship between LC9 and CKD using restricted cubic spline **(B)**. The solid line represents the fitted nonlinearcurve. The area adjacent to the solid line represents the 95% confidence Interval. LC9, Life’s Crucial 9; CKD, Chronic kidney disease; CI, confidence interval.

A histogram was used to visualize the distribution of LC9 (Figure 2A). To evaluate the potential nonlinear relationship between LC9 and CKD, restricted cubic spline (RCS) analysis was employed. *p*-values for nonlinearity were calculated to assess statistical significance. Due to the high collinearity between LC9 and LE8 (VIF = 26), we avoided including both variables in the same regression model for comparison. We used receiver operating characteristic (ROC) curve analysis to compare their predictive accuracy for CKD risk, as this method is not affected by multicollinearity. Z-tests were used to determine whether the predictive performance of LC9 and LE8 significantly differed.

**Figure 3:**
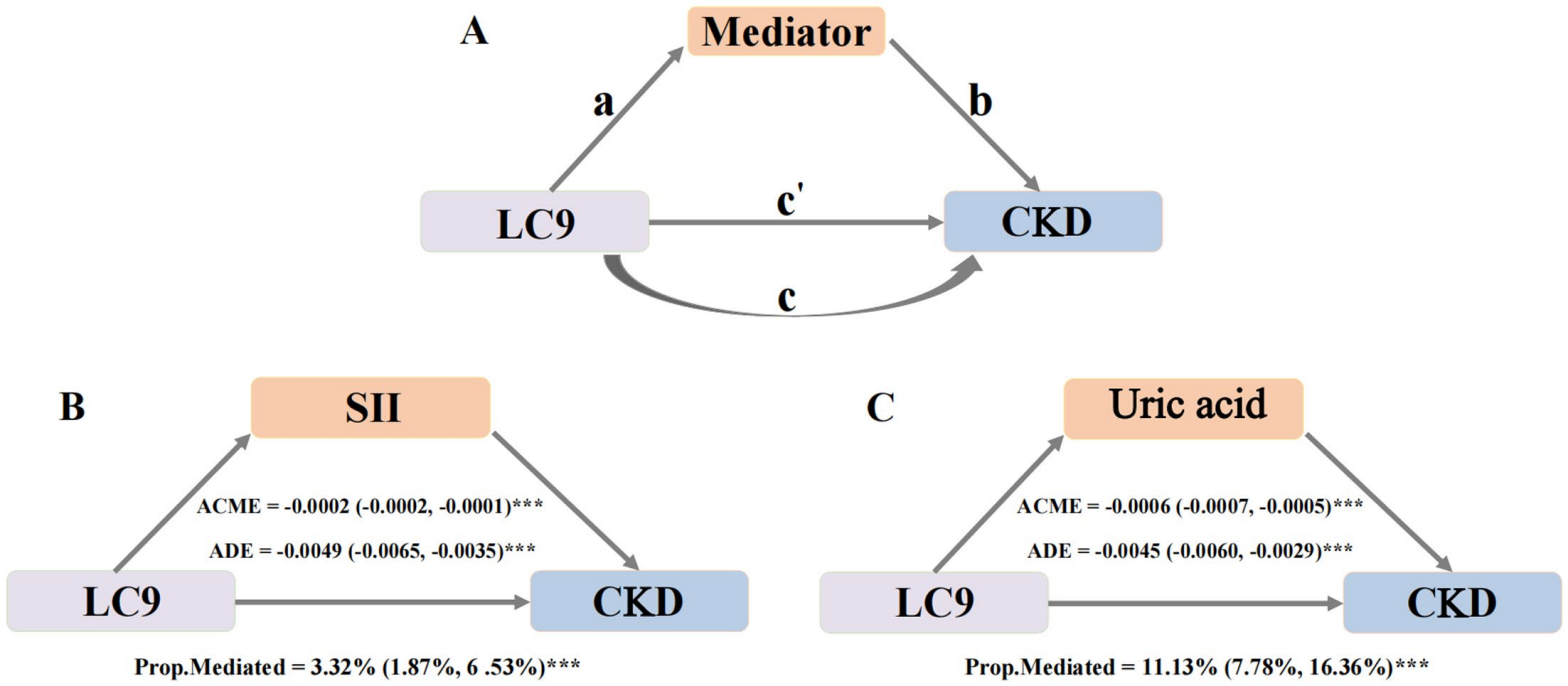
The modified caption is as follows: Mediation effects of potential mediators in the associations of LC9 with CKD. Panel A shows the general mediation model. Path a represents the association between LC9 and the mediators; path b represents the effect of the mediators on CKD after adjusting for LC9; path c is the direct effect of LC9 on CKD after adjusting for the mediators; path c is the total effect of LC9 on CKD without accounting for the mediators. The Indirect effect is calculated as a x b. Mediation proportion Indirect effect/total effect. Panel B illustrates the mediating effect of SII. Panel C demonstrates the mediating role of Uric acid. Adjust for age, sex, race, body mass index, poverty income ratio, education levels, marital status, smoking status, alcohol consumption, PA total MET, hyperlipidemia, hypertension, diabetes mellitus and cardiovascular disease. CKD, Chronic kidney disease; LC9, Life’s Crucial 9; SII, Systemic Immune-inflammation index; ACME, Average causal mediation effects (Indirect effect); ADE, Average direct effects. *p < 0.005, **p < 0.01, and ***p < 0.001.

A mediation analysis was performed using the “Mediation” package in R. As depicted in Figure 3A, the analysis followed a two-step approach. (1) Path a: regression models were used to assess the effect of LC9 on the mediators. (2) Next, after accounting for the mediators, mediators’ impact on CKD (path b) was assessed, along with the effect of LC9 on CKD (path c’). Indirect effect = path a*path b. The mediation proportion = indirect effect/total effect. The total effect of LC9 on CKD was estimated without considering for mediators (path c). A bootstrap method with 500 iterations was applied to generate 95% confidence intervals for the mediation proportion, ensuring robust estimates.

## 3 Results

### 3.1 Descriptive characteristics

A total of 16,431 participants were included in this study (Table 1). The characteristics of the study population were stratified based on the presence or absence of CKD. The prevalence of CKD significantly increased with age (*p* < 0.001), with the highest prevalence observed among participants aged ≥ 60 years (24.38%). Among the study population, 7,867 (48.28%) were female, and 8,564 (51.72%) were male. The prevalence of CKD was significantly higher among females (12.18%) compared to males (9.53%, *p* < 0.001). In terms of race, non-Hispanic Black individuals had the highest prevalence of CKD (14.13%), while Hispanics had the lowest prevalence (8.66%). BMI was also strongly associated with CKD prevalence (*p* < 0.001); CKD was more frequent in participants categorized as obese (13.55%), compared to those with overweight (9.40%) and underweight/normal (9.26%).

Socioeconomic factors were also significantly linked to CKD prevalence. The highest prevalence was found in participants who were widowed, divorced, or separated (18.68%), compared to those who were married/living with a partner (10.19%) or never married (6.34%) (*p* < 0.001). Education level was inversely associated with CKD prevalence, with participants having below high school education showing the highest prevalence (17.60%, *p* < 0.001). Regarding lifestyle factors, former smokers (13.47%) had a higher prevalence of CKD compared to never smokers (10.00%) and current smokers (9.79%, *p* < 0.001). Former alcohol consumers had the highest CKD prevalence (16.80%).

Participants with hyperlipidemia (12.58%), hypertension (19.89%), diabetes mellitus (28.93%), and cardiovascular disease (31.43%) had significantly higher CKD prevalence compared to those without these conditions (*p* < 0.001 for all). In addition, individuals with CKD had lower LC9 and LE8 scores compared to those without CKD (*p* < 0.001), with mean LC9 scores of 68.73 ± 0.54 vs. 74.69 ± 0.23, and mean LE8 scores of 66.44 ± 0.55 vs. 72.70 ± 0.24. Participants with CKD also had higher levels of uric acid, SII, and SIRI, along with lower physical activity total MET (PA total MET) values compared to those without CKD (*p* < 0.001 for all).

### 3.2 Binary logistic regression analysis

Binary logistic regression was performed to assess the association between LC9 score and CKD, with results summarized in Table 2. In the unadjusted model, LC9 (as a continuous variable) demonstrated a significant inverse association with CKD (OR: 0.96, 95% CI: 0.96–0.97, *p* < 0.001). This association remained robust in Model 1 (OR: 0.97, 95% CI: 0.96–0.97, *p* < 0.001) and persisted in Model 2 (OR: 0.98, 95% CI: 0.97–0.99, *p* < 0.001). When LC9 was analyzed in quartiles (Q1–Q4), in the fully adjusted Model 2, participants in the highest quartile (Q4) had significantly lower odds of CKD compared to those in the lowest quartile (Q1) (OR: 0.57, 95% CI: 0.38–0.85, *p* = 0.01). Similarly, those in Q3 (OR: 0.58, 95% CI: 0.44–0.77, *p* < 0.001) and Q2 (OR: 0.72, 95% CI: 0.59–0.89, *p* = 0.003) also exhibited significantly lower odds of CKD relative to Q1. The *P* for trend across quartiles remained statistically significant in all models (*p* < 0.001 in the unadjusted and Model 1; *p* = 0.002 in Model 2), further reinforcing the graded relationship between higher LC9 scores and reduced CKD risk.

**Table 2:** Adjusted association of LC9 with CKD.

Exposure	Unadjusted model	Adjust 1	Adjust 2
	Odds ratio (95% CI) associated with CKD		
LC9 (continuous)	0.96 (0.96, 0.97); **< 0.001**	0.97 (0.96, 0.97); **< 0.001**	0.98 (0.97, 0.99); **< 0.001**
Quartile of LC9			
Q1	1 (Ref)	1 (Ref)	1 (Ref)
Q2	0.58 (0.50, 0.68); **< 0.001**	0.57 (0.48, 0.67); **< 0.001**	0.72 (0.59, 0.89); **0.003**
Q3	0.39 (0.33, 0.46); **< 0.001**	0.40 (0.33, 0.49); **< 0.001**	0.58 (0.44, 0.77); **< 0.001**
Q4	0.29 (0.23, 0.36); **< 0.001**	0.34 (0.27, 0.44); **< 0.001**	0.57 (0.38, 0.85); **0.01**
*P* for trend	**< 0.001**	**< 0.001**	**0.002**

Unadjusted model: non-adjusted model.

Adjust 1: Adjust for age, sex, race.

Adjust 2: Adjust for age, sex, race, body mass index, poverty income ratio, education levels, marital status, smoking status, alcohol consumption, PA total MET, hyperlipidemia, hypertension, diabetes mellitus and cardiovascular disease.

CKD, Chronic kidney disease; LC9, Life’s Crucial 9; CI, Confidence interval. Bold values indicate statistical significance at *P* < 0.05.

### 3.3 Subgroup analyses and sensitivity analysis

The results of the subgroup analyses are presented in Table 3. Overall, the inverse association between LC9 and CKD remained consistent across most subgroups. However, significant interactions were observed for sex (*P* for interaction = 0.044), age (*p* = 0.008), and BMI categories (*p* = 0.014). These findings suggest that the association between LC9 and CKD may be more pronounced in certain groups, particularly females, middle-aged adults (30–59 years), and individuals with overweight or obesity.

**Table 3:** Adjusted association of LC9 with CKD for subgroup analyses.

Subgroups	Adjusted odds ratio (95% confidence interval); p*	*P* for interaction
Sex		**0.044**
Female	0.972 (0.957, 0.988); **< 0.001**	
Male	0.981 (0.966, 0.996); **0.015**	
Age		**0.008**
18–29	0.984 (0.949, 1.019); 0.355	
30–44	0.965 (0.947, 0.984); **< 0.001**	
45–59	0.973 (0.950, 0.996); **0.025**	
> =60	0.986 (0.972, 1.001); 0.070	
Body mass index		**0.014**
Underweight/Normal	0.987 (0.964, 1.010); 0.271	
Overweight	0.976 (0.958, 0.995); **0.013**	
Obese	0.973 (0.959, 0.987); **< 0.001**	
Smoking status		0.576
Never	0.972 (0.958, 0.985); **< 0.001**	
Former	0.983 (0.963, 1.004); 0.104	
Now	0.986 (0.960, 1.014); 0.317	
Hyperlipidemia		0.084
No	0.976 (0.963, 0.989); **< 0.001**	
Yes	0.989 (0.965, 1.013); 0.366	
Hypertension		0.133
No	0.970 (0.955, 0.984); < **0.001**	
Yes	0.989 (0.973, 1.005); 0.166	
Diabetes mellitus		0.066
No	0.983 (0.966, 1.001); 0.057	
Prediabetes	1.003 (0.975, 1.031); 0.850	
DM	0.955 (0.941, 0.971); **< 0.001**	
Cardiovascular disease		0.239
No	0.990 (0.960, 1.021); 0.509	
Yes	0.975 (0.962, 0.988); **< 0.001**	

^*^Adjust for age, sex, race, body mass index, poverty income ratio, education levels, marital status, smoking status, alcohol consumption, hyperlipidemia, hypertension, diabetes mellitus, triglyceride, high density lipoprotein and PA total MET, but not for the specific stratification variables of interest. Bold values indicate statistical significance at *P* < 0.05.

Several possible explanations may underlie these subgroup differences. First, women may exhibit greater physiological responsiveness to cardiovascular health interventions due to sex-specific hormonal or inflammatory profiles, which may enhance the protective impact of LC9 (37). Second, middle-aged adults are often at a critical stage for both cardiovascular and renal risk accumulation, yet still within a window where lifestyle modifications could yield meaningful benefit. Third, individuals with overweight or obesity experience amplified metabolic stress and inflammation, making them more susceptible to CKD but also more likely to benefit from improvements in LC9 components such as diet, PA, and mental health (38).

To test the robustness of the findings, sensitivity analyses were performed using an alternative model without applying survey weights (Supplementary Table S3). The results remained largely consistent with the primary analyses, confirming that higher LC9 scores were significantly associated with lower odds of CKD across all models.

In summary, subgroup and sensitivity analyses consistently supported the protective association between LC9 and CKD, with variations observed across specific subpopulations. These results highlight the potential role of LC9 in CKD prevention and underscore its applicability across diverse demographic and clinical groups.

### 3.4 Nonlinear relationships explore

The RCS analysis revealed a statistically significant nonlinear relationship (nonlinearity *p* < 0.001), suggesting that the association between LC9 and CKD does not follow a strictly linear pattern when using four knots (Figure 2). To further test the robustness of this finding, we conducted sensitivity analyses by varying the number of knots from 3 to 8 (Supplementary Table S4). Across all knot selections, the nonlinearity *p*-values remained significant (all *p* < 0.001), reinforcing the presence of a nonlinear association.

### 3.5 Mediation analysis

Mediation analysis was conducted to assess whether the relationship between LC9 and CKD was partially explained by systemic inflammation and oxidative stress. In this analysis, LC9 served as the independent variable, CKD as the dependent variable, and selected potential mediators as mediator variables.

First, we examined the association between LC9 and potential mediators (Supplementary Table S5). After full adjustment, SII, bilirubin and uric acid showed significant associations with LC9, suggesting that path a (the effect of LC9 on the mediator) was significant for these factors. Next, the association between potential mediators and CKD was analyzed while controlling for LC9 and all covariates (Supplementary Table S6). The results indicated that both SII, SIRI and uric acid were significantly linked to CKD, confirming the presence of path b (the effect of the mediator on CKD). However, bilirubin did not show a significant relationship with CKD and was therefore excluded from further mediation analysis.

As both path a and path b were statistically significant for SII and uric acid, these two mediators were selected for final mediation modeling (Figure 3). The analysis revealed significant indirect effects of LC9 on CKD through these mediators. SII mediated 3.32% (95% CI: 1.87–6.53%, *p* < 0.001) of the total effect. Uric acid mediated 11.13% (95% CI: 7.78–16.36%, *p* < 0.001). These findings indicate that systemic inflammation and oxidative stress contribute to the association between LC9 and CKD, reinforcing the role of systemic inflammation and uric acid levels in kidney health.

### 3.6 Comparing the LC9 and LE8

**Figure 4:**
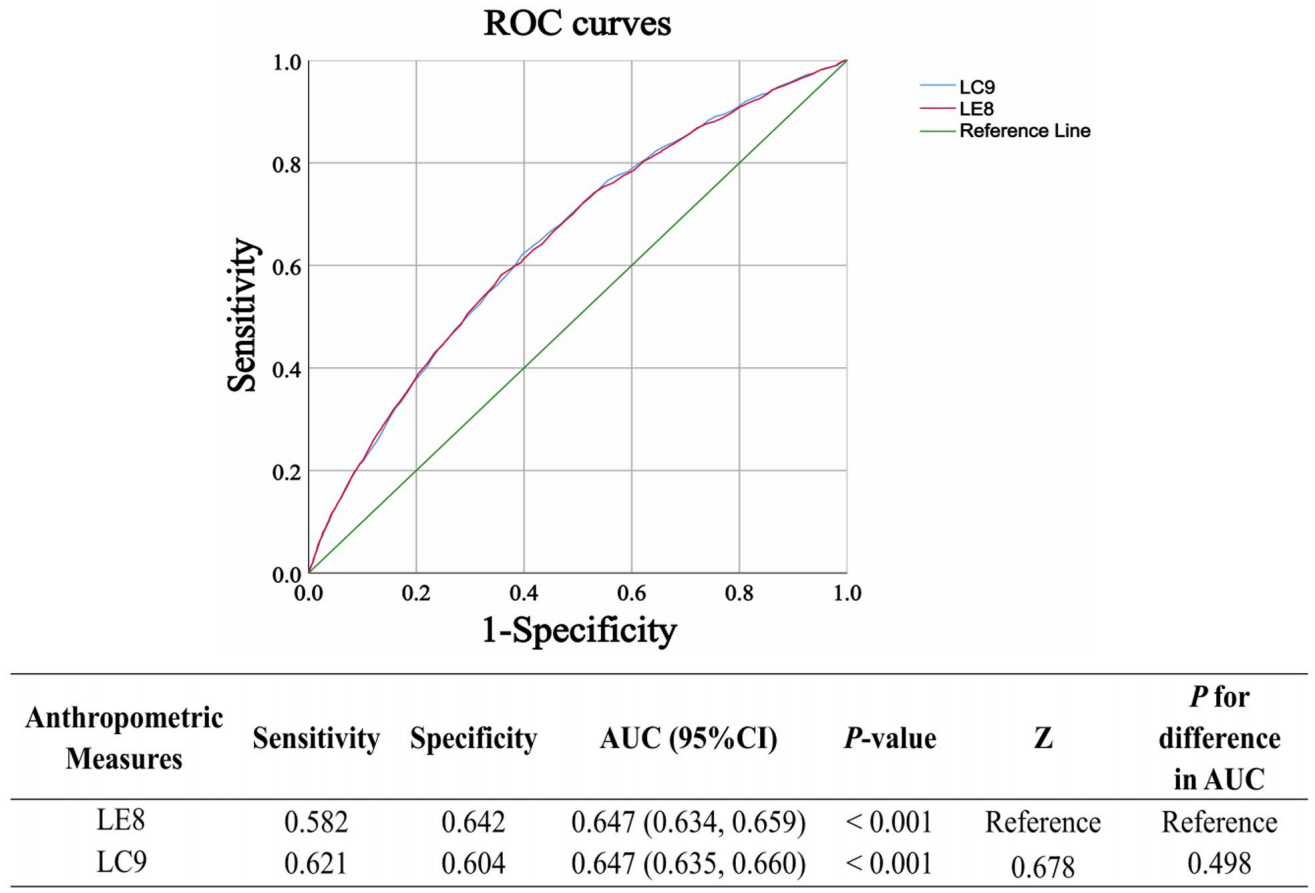
The receiver operating characteristic (ROC) curves for the prediction of CKD using LC9 and LE8 are presented. The Z-test of the area under the ROC curve (AUC) is used to assess the significant difference in predictive performance between the prediction models. LC9, Life’s Crucial 9; LE8, Life’s Essential 8.

To assess the predictive performance of LC9 and LE8 for CKD, ROC curves were generated (Figure 4). The Z-test of the area under the curve (AUC) was used to compare the two models. The results indicated no statistically significant difference between the predictive abilities of LC9 and LE8 (*p* = 0.498), suggesting that both indices have similar performance in identifying individuals at risk for CKD.

## 4 Discussion

This study comprehensively investigated the association between LC9 and CKD, examining its predictive performance relative to LE8 and exploring potential mediating pathways. Our findings highlight the significant inverse association between LC9 and CKD, with a nonlinear relationship, and demonstrate that systemic inflammation and uric acid levels partially mediate this association. Additionally, we found that while LC9 and LE8 demonstrated similar predictive abilities for CKD.

Our analysis identified distinct patterns in CKD prevalence across socio-demographic groups. Older age, female sex, obesity, lower educational attainment, and lower socioeconomic status were associated with a higher prevalence of CKD. These findings align with previous studies analyzing national datasets, which have emphasized the influence of social determinants and health disparities on kidney disease risk (8, 30, 39). These results highlight the importance of considering these covariates when evaluating the association between LC9 and CKD. Moreover, our findings demonstrate that CKD is more prevalent in individuals with hyperlipidemia, hypertension, DM and CVD, conditions that are well-established risk factors for renal dysfunction and cardiovascular complications (10, 40–42). Hyperlipidemia has been strongly linked to glomerular injury, with studies showing that elevated lipid levels accelerate CKD progression and worsen renal outcomes (40). Similarly, hypertension is a major independent risk factor for CKD, as increased blood pressure contributes to glomerular hypertension and nephron loss, even in individuals without other metabolic disorders (43). DM and CKD are closely linked, with DM being the leading cause of kidney failure worldwide, driven by shared metabolic and cardiovascular risk factors (42). CKD and CVD are strongly interconnected, with shared common risk factors such as hypertension, diabetes, and dyslipidemia (10).

Discussing the relationship between depression and CKD is essential, as LC9 differs from LE8 by incorporating mental health factors, recognizing that depression not only coexists with CKD but also influences its progression through behavioral and biological mechanisms. Depression and CKD share a bidirectional relationship, with depressive symptoms increasing the risk of CKD onset and progression, while impaired renal function elevates the likelihood of developing depression (44). Individuals with depression often exhibit poor health behaviors such as physical inactivity, smoking, and unhealthy dietary patterns, which are also recognized risk factors for CKD (44–46). Additionally, chronic inflammation and dysregulation of the hypothalamic–pituitary–adrenal (HPA) axis in depression may contribute to intrarenal microcirculatory dysfunction, endothelial damage, and accelerated renal decline (46–48). Conversely, CKD-associated systemic inflammation and oxidative stress may impair neurotransmitter function and increase neuropsychiatric symptoms, fostering the onset of depression (48–51). These findings emphasize the need for early screening and management of depression in CKD patients and vice versa, as addressing both conditions concurrently may help mitigate their detrimental effects on overall health.

One of the novel contributions of this study is the identification of systemic inflammation and uric acid levels as mediators in the LC9-CKD relationship. Inflammation plays a critical role in the development and progression of CKD, contributing to renal fibrosis, endothelial dysfunction, and accelerated decline in kidney function (52). Persistent low-grade inflammation in CKD is driven by an imbalance between pro-and anti-inflammatory factors, resulting from immune system dysregulation, oxidative stress, gut dysbiosis, and impaired clearance of inflammatory mediators (52). Key inflammatory markers, including TNF-*α*, IL-6, and IL-1β, are elevated in CKD and are associated with worsening renal outcomes and increased cardiovascular risk (52, 53). Furthermore, inflammasomes such as NLRP3 contribute to CKD progression by promoting pro-inflammatory cytokine release and renal injury (54). The inflammatory burden in CKD is further exacerbated by dialysis-related immune activation, highlighting the need for targeted interventions to mitigate inflammation and slow disease progression (55). Uric acid is increasingly recognized as a key player in CKD progression, contributing through multiple pathogenic mechanisms (33). Hyperuricemia has been linked to renal vasoconstriction, endothelial dysfunction, oxidative stress, and chronic inflammation, all of which accelerate glomerulosclerosis and tubulointerstitial fibrosis (33). Elevated uric acid levels promote the activation of the renin–angiotensin–aldosterone system (RAAS), leading to increased blood pressure and reduced renal perfusion, which further exacerbates CKD progression (33, 56). Additionally, uric acid stimulates pro-inflammatory cytokine production and impairs nitric oxide bioavailability, contributing to vascular damage and kidney injury (57). The dual role of uric acid in oxidative stress is particularly relevant, as it can function as both an antioxidant extracellularly and a pro-oxidant intracellularly, with excessive uric acid levels favoring oxidative damage and renal impairment (33, 57, 58). These findings highlight the need for targeted strategies to manage uric acid levels in CKD patients, with emerging evidence suggesting that uric-acid-lowering therapies may help slow disease progression (33).

Our findings have several important implications for CKD prevention and management. Since LC9 encompasses both lifestyle behaviors and metabolic health indicators, it provides a holistic approach to CKD risk assessment. Interventions focusing on diet, physical activity, smoking cessation, metabolic health optimization and mental health may collectively reduce CKD burden. Given the mediation effects of systemic inflammation and uric acid, strategies that reduce chronic inflammation and improve metabolic health—such as anti-inflammatory dietary patterns and uric acid-lowering therapies—may be effective in preventing CKD progression. Future studies should refine risk prediction models by integrating LC9 components into CKD screening protocols.

This study has several methodological strengths: The use of a large, nationally representative dataset (NHANES) enhances generalizability to diverse U.S. populations. The incorporation of RCS analysis, mediation models, and ROC analysis provides robust analytical depth. The study comprehensively examined metabolic and inflammatory pathways, contributing to a more integrated understanding of CKD risk factors. However, several limitations should be acknowledged. First and most importantly, the cross-sectional design of this study limits the ability to establish causal relationships. The reliance on self-reported data for lifestyle behaviors introduces potential recall bias. While LC9 and LE8 capture essential health components, additional risk factors (e.g., gut microbiota, genetic predisposition) were not included in this study and warrant further exploration (59, 60). Moreover, important covariates such as medication use and receipt of treatment for mental health conditions were not adjusted for in our models. Specifically, although NHANES includes data on antidepressant medication use, the complexity of extraction and potential for misclassification prevented its inclusion. Furthermore, psychotherapy status was not available in NHANES.

Future longitudinal cohort studies are needed to clarify the causal relationship between LC9 and CKD, and to assess whether changes in LC9 components over time are associated with kidney function outcomes. Given that LC9 primarily reflects cardiovascular health, it is particularly important to investigate CKD risk among individuals with existing cardiovascular risk factors. Future prospective research should explore whether targeted interventions aimed at improving LC9 scores in high-risk populations, such as those with hypertension, diabetes, or established cardiovascular disease, can improve cardiovascular outcomes while also reducing the onset or progression of CKD. In addition, well-designed intervention trials focusing on modifiable components of LC9, including diet, physical activity, and mental health, may help establish causal relationships and provide actionable evidence for CKD prevention.

## 5 Conclusion

In conclusion, this study highlights the inverse association between LC9 and CKD, with systemic inflammation and uric acid levels mediating part of this relationship. LC9 and LE8 exhibited similar predictive performance of CKD risk. These findings support the adoption of comprehensive lifestyle interventions targeting the nine components of LC9—diet quality, physical activity, nicotine exposure, sleep duration, body weight management, blood glucose control, blood pressure regulation, lipid profile, and depression—as well as addressing systemic inflammation and oxidative stress to reduce the burden of CKD. However, due to the cross-sectional design of this study, these findings should be interpreted as associations rather than causal relationships.

## Data Availability

The datasets presented in this study can be found in online repositories. The names of the repository/repositories and accession number(s) can be found in the article/Supplementary material.
