# Supplementary material for: Life’s Crucial 9 score and chronic kidney disease: insights from NHANES 2005–2018 and the mediating role of systemic inflammation and oxidative stress
Source: Front Med (Lausanne). 2025 Jun 18;12:1605931. doi: 10.3389/fmed.2025.1605931 (PMC12213831; doi:10.3389/fmed.2025.1605931)
Supplement: Supplementary file 2 [file Table_2.docx]

| **Variables** | **Division details** | **Corresponding range** | **Frequency** | **Percentage** |
| --- | --- | --- | --- | --- |
| **LC9** | Q1 | [16.111, 64.444] | 4007 | 24.39% |
|  | Q2 | (64.444, 73.333] | 4161 | 25.32% |
|  | Q3 | (73.333, 81.667] | 4311 | 26.24% |
|  | Q4 | (81.667, 100] | 3952 | 24.05% |
| **LE8** | Q1 | [18.125, 61.875] | 4264 | 25.95% |
|  | Q2 | (61.875,70.625] | 3960 | 24.10% |
|  | Q3 | (70.625,80] | 4221 | 25.69% |
|  | Q4 | (80,100] | 3986 | 24.26% |

**Table S2**. Details of LC9 and LE8 division.
