# Supplementary material for: Life’s Crucial 9 score and chronic kidney disease: insights from NHANES 2005–2018 and the mediating role of systemic inflammation and oxidative stress
Source: Front Med (Lausanne). 2025 Jun 18;12:1605931. doi: 10.3389/fmed.2025.1605931 (PMC12213831; doi:10.3389/fmed.2025.1605931)
Supplement: Supplementary file 3 [file Table_3.docx]

**Table S3*.*** Adjusted association of LC9 with CKD for sensitivity analysis.

| **Exposure** | **Unadjusted model** | **Adjust 1** | **Adjust 2** |
| --- | --- | --- | --- |
|  | Odds ratio (95% CI) associated with CKD | | |
| **LC9 (continuous)** | 0.96 (0.96, 0.96); **< 0.001** | 0.96 (0.96, 0.97); **< 0.001** | 0.98 (0.97, 0.98); **< 0.001** |
| **Quartile of LC9** |  |  |  |
| Q1 | 1 (Ref) | 1 (Ref) | 1 (Ref) |
| Q2 | 0.61 (0.55, 0.68); **< 0.001** | 0.60 (0.54, 0.67); **< 0.001** | 0.74 (0.66, 0.83); **< 0.001** |
| Q3 | 0.40 (0.35, 0.47); **< 0.001** | 0.43 (0.37, 0.49); **< 0.001** | 0.60 (0.50, 0.71); **< 0.001** |
| Q4 | 0.25 (0.21, 0.29); **< 0.001** | 0.33 (0.28, 0.40); **< 0.001** | 0.53 (0.42, 0.68); **< 0.001** |
| *P* for trend | **< 0.001** | **< 0.001** | **< 0.001** |

Unadjusted model: non-adjusted model.

Adjust 1: Adjust for age, sex, race.

Adjust 2: Adjust for age, sex, race, body mass index, poverty income ratio, education levels, marital status, smoking status, alcohol consumption, PA total MET, hyperlipidemia, hypertension, diabetes mellitus and cardiovascular disease.

**Abbreviations**: CKD, Chronic kidney disease; LC9, Life's Crucial 9; CI, Confidence interval.
