# Supplementary material for: Life’s Crucial 9 score and chronic kidney disease: insights from NHANES 2005–2018 and the mediating role of systemic inflammation and oxidative stress
Source: Front Med (Lausanne). 2025 Jun 18;12:1605931. doi: 10.3389/fmed.2025.1605931 (PMC12213831; doi:10.3389/fmed.2025.1605931)
Supplement: Supplementary file 4 [file Table_4.docx]

**Table S4*.*** Nonlinear *P*-values of LC9 and CKD at different knots.

| **LC9 knots** | **Nonlinear P-values** |
| --- | --- |
| 3 | 0.000050 |
| 4 | 0.000029 |
| 5 | 0.000120 |
| 6 | 0.000262 |
| 7 | 0.000526 |
| 8 | 0.000021 |

**Abbreviations**: CKD, Chronic kidney disease; LC9, Life's Crucial 9.
