# Supplementary material for: Life’s Crucial 9 score and chronic kidney disease: insights from NHANES 2005–2018 and the mediating role of systemic inflammation and oxidative stress
Source: Front Med (Lausanne). 2025 Jun 18;12:1605931. doi: 10.3389/fmed.2025.1605931 (PMC12213831; doi:10.3389/fmed.2025.1605931)
Supplement: Supplementary file 5 [file Table_5.docx]

**Table S5*.*** Relationship between LC9 and potential mediators in different models.

| **Outcomes** | **Unadjusted model** | | **Adjust 1** | | **Adjust 2** |
| --- | --- | --- | --- | --- | --- |
|  | β (95% CI ) associated with potential mediating variables | | | | |
| **SII** | -2.42 (-2.95, -1.88); **< 0.001** | -2.89 (-3.43, -2.34); **< 0.001** | | -1.82 ( -2.71, -0.93); **< 0.001** | |
| **SIRI** | -0.01 (-0.01, -0.01); **< 0.001** | -0.01 (-0.01, -0.01); **< 0.001** | | 0 (0.00, 0.00); 0.12 | |
| **Bilirubin*** | 0 (0.00, 0.00); **< 0.001** | 0 (0.00, 0.00); **< 0.001** | | 0 (0.00, 0.00); **< 0.001** | |
| **Uric acid*** | -0.03 (-0.03, -0.03); **< 0.001** | -0.02 (-0.03, -0.02); **< 0.001** | | -0.01 (-0.01, -0.01); **< 0.001** | |

* Bilirubin and uric acid were expressed in units of mg/dL.

Unadjusted model: non-adjusted model.

Adjust 1: Adjust for age, sex, race.

Adjust 2: Adjust for age, sex, race, body mass index, poverty income ratio, education levels, marital status, smoking status, alcohol consumption, PA total MET, hyperlipidemia, hypertension, diabetes mellitus and cardiovascular disease.

**Abbreviations**: CKD, Chronic kidney disease; LC9, Life's Crucial 9; METS-IR, [metabolic score for insulin resistance;](https://link.springer.com/article/10.1186/s12933-024-02334-8) HOMA-IR, homeostatic model assessment of insulin resistance; SII, systemic immune-inflammation index; SIRI, systemic inflammation response index; GGT, serum gamma-glutamyltransferase; CI, confidence interval.
