# Supplementary material for: Life’s Crucial 9 score and chronic kidney disease: insights from NHANES 2005–2018 and the mediating role of systemic inflammation and oxidative stress
Source: Front Med (Lausanne). 2025 Jun 18;12:1605931. doi: 10.3389/fmed.2025.1605931 (PMC12213831; doi:10.3389/fmed.2025.1605931)
Supplement: Supplementary file 6 [file Table_6.docx]

**Table S6*.*** Relationship between LC9 and potential mediators with CKD in different models.

| **Exposure** | | **Unadjusted model** | **Adjust 1** | **Adjust 2** |
| --- | --- | --- | --- | --- |
|  |  | Odds ratio (95% CI) associated with CKD | | |
| **Exposure 1** | **LC9** | 0.96 (0.96, 0.97); **< 0.001** | 0.97 (0.96, 0.97); **< 0.001** | 0.98 (0.97, 0.99); **< 0.001** |
|  | **SII** | 1.00 (1.00, 1.00); **< 0.001** | 1.00 (1.00, 1.00); **< 0.001** | 1.00 (1.00, 1.00); **< 0.001** |
| **Exposure 2** | **LC9** | 0.96 (0.96, 0.97); **< 0.001** | 0.97 (0.96, 0.97); **< 0.001** | 0.98 (0.97, 0.99); **< 0.001** |
|  | **SIRI** | 1.31 (1.20, 1.43); **< 0.001** | 1.29 (1.19, 1.39); **< 0.001** | 1.23 (1.14, 1.34); **< 0.001** |
| **Exposure 3** | **LC9** | 0.96 (0.96, 0.97); **< 0.001** | 0.97 (0.96, 0.97); **< 0.001** | 0.98 (0.97, 0.99); **< 0.001** |
|  | **Bilirubin*** | 0.98 (0.80, 1.21); 0.88 | 1.12 (0.88, 1.43); 0.34 | 1.05 (0.82, 1.35); 0.92 |
| **Exposure 4** | **LC9** | 0.97 (0.96, 0.97); **< 0.001** | 0.97 (0.96, 0.98); **< 0.001** | 0.98 (0.97, 0.99); **0.001** |
|  | **Uric acid*** | 1.21 (1.14, 1.28); **< 0.001** | 1.32 (1.24, 1.41); **< 0.001** | 1.35 (1.26, 1.45); **< 0.001** |

* Bilirubin and uric acid were expressed in units of mg/dL.

Unadjusted model: non-adjusted model.

Adjust 1: Adjust for age, sex, race.

Adjust 2: Adjust for age, sex, race, body mass index, poverty income ratio, education levels, marital status, smoking status, alcohol consumption, PA total MET, hyperlipidemia, hypertension, diabetes mellitus and cardiovascular disease.

**Abbreviations**: CKD, Chronic kidney disease; LC9, Life's Crucial 9; METS-IR, [metabolic score for insulin resistance;](https://link.springer.com/article/10.1186/s12933-024-02334-8) HOMA-IR, homeostatic model assessment of insulin resistance; SII, systemic immune-inflammation index; SIRI, systemic inflammation response index; GGT, serum gamma-glutamyltransferase; CI, confidence interval.
