# Supplementary figures and images for: Life’s Crucial 9 score and chronic kidney disease: insights from NHANES 2005–2018 and the mediating role of systemic inflammation and oxidative stress
Source: Front Med (Lausanne). 2025 Jun 18;12:1605931. doi: 10.3389/fmed.2025.1605931 (PMC12213831; doi:10.3389/fmed.2025.1605931)

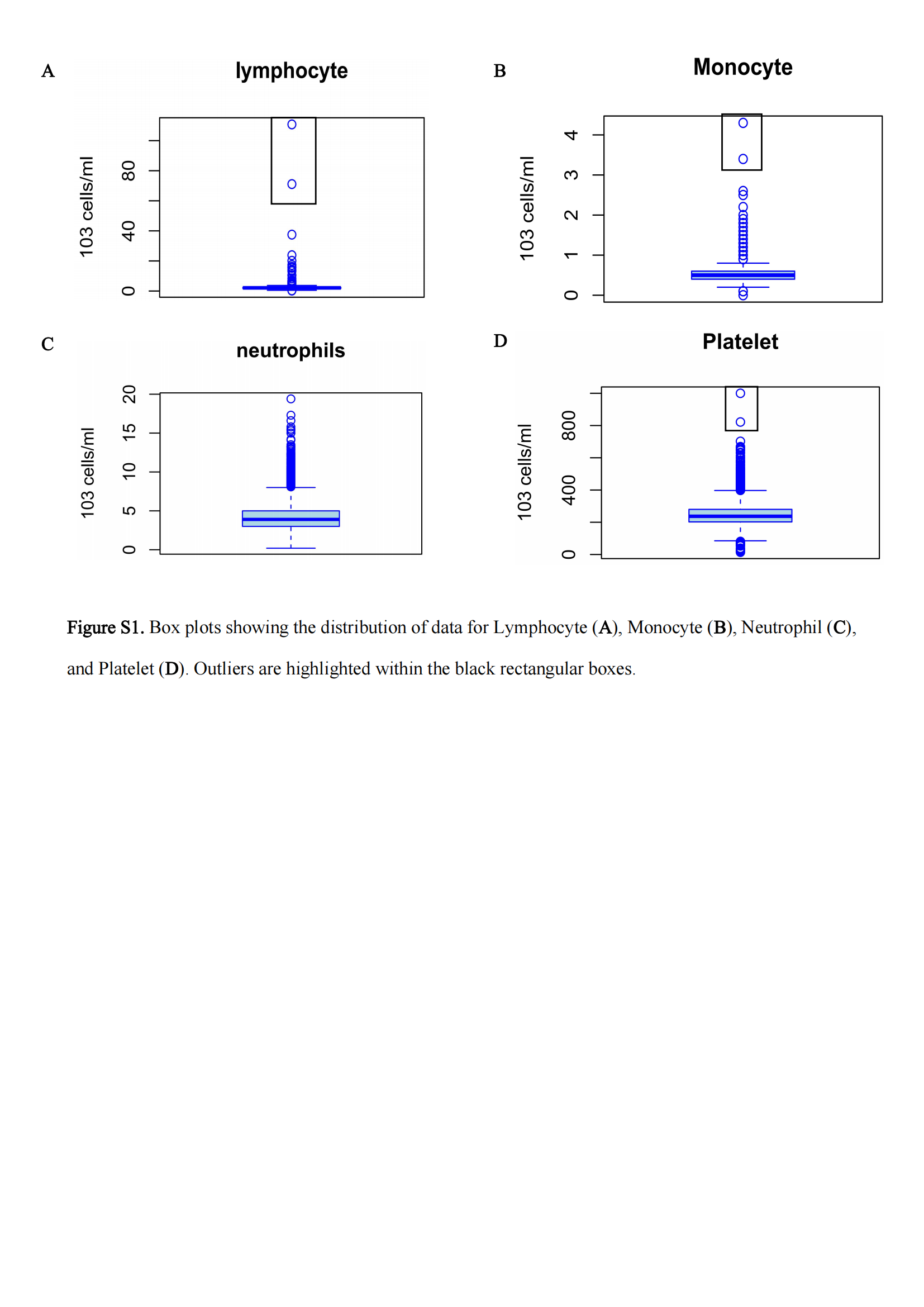

Supplement: Supplementary file 7 [file Image_1.tif]

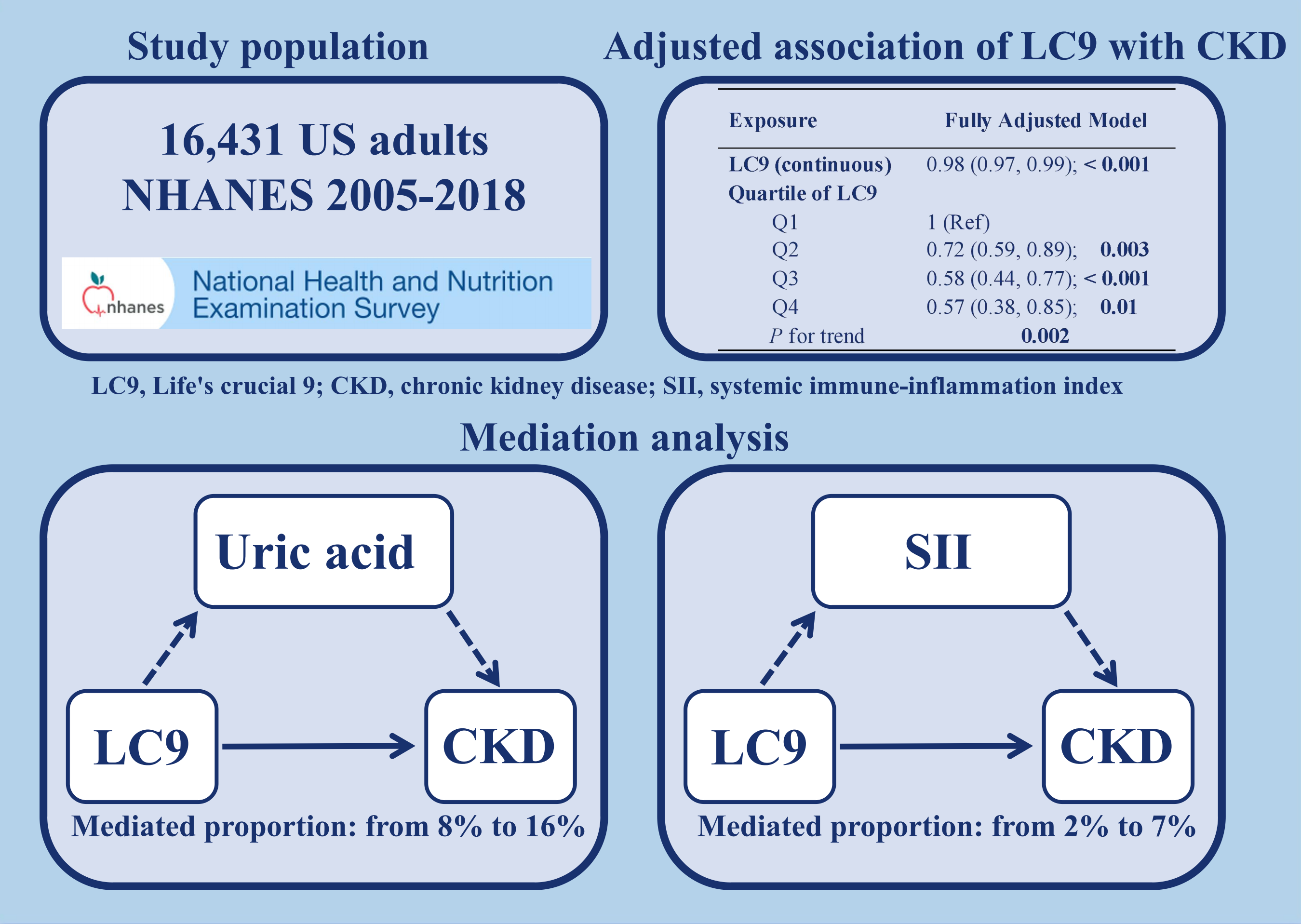

Supplement: Supplementary file 8 [file Image_2.jpeg]
